# Lung cancer in never smokers from the Princess Margaret Cancer Centre

**DOI:** 10.18632/oncotarget.25176

**Published:** 2018-04-27

**Authors:** Grzegorz J. Korpanty, Suzanne Kamel-Reid, Melania Pintilie, David M. Hwang, Alona Zer, Geoffrey Liu, Natasha B. Leighl, Ronald Feld, Lillian L. Siu, Philippe L. Bedard, Ming-Sound Tsao, Frances A. Shepherd

**Affiliations:** ^1^ Princess Margaret Cancer Centre, University Health Network, Toronto, ON, Canada; ^2^ Laboratory Genetics, University Health Network, Toronto, ON, Canada; ^3^ Laboratory Medicine and Pathobiology, University of Toronto, Toronto, ON, Canada

**Keywords:** lung cancer, never smokers, targeted therapy

## Abstract

**Introduction:**

Lung cancer in never smokers represents a distinct epidemiological, clinical, and molecular entity.

**Results:**

Most 712 never smoking lung cancer patients were female (72%) with a median age at diagnosis of 62.2 years (18–94). Caucasians (46%), East Asians (42%), adenocarcinoma histology (87%) and presentation with metastatic disease at diagnosis (59%) were common. Of 515 patients with available archival tissue, the most common identified single mutations were *EGFR* (52.2%), followed by *ALK* (7.5%), *KRAS* (2.3%), *TP53* (1.3%), *ERBB2* (1%), *BRAF* (0.4%), *PIK3CA* (0.4%), *SMAD4* (0.4%), *CTNNB1* (0.2%), *AKT1* (0.2%), and *NRAS* (0.2%); 8% tumors had multiple mutations, while 25.8% had none identified. Median overall survival (mOS) was 42.2 months (mo) for the entire cohort. Patients with mutations in their tumors had significantly better mOS (69.5 mo) when compared to those without (31.0 mo) (HR = 0.59; 95% CI: 0.44–0.79; *p* < 0.001). Earlier stage (*p* < 0.001), adenocarcinoma histology (*p* = 0.012), good performance status (*p* < 0.001) and use of targeted therapy (*p* < 0.001) were each independently associated with longer survival. Patients with *ALK*-translocation-positive tumours have significantly longer OS compared to those without any mutations (*p* = 0.0029) and to those with other and null mutations (*p* = 0.022).

**Conclusions:**

Lung cancer in never smokers represents a distinct clinical and molecular entity characterized by a high incidence of targetable mutations and long survival.

**Methods:**

We analyzed retrospectively the data from electronic patient records of never smokers diagnosed with lung cancer treated at the Princess Margaret Cancer Centre (Toronto) between 1988–2015 to characterize demographic and clinical features, pathology, molecular profile (using hotspot or targeted sequencing panels), treatment and survival.

## INTRODUCTION

Although 80–90% of lung cancer cases are associated with inhaled tobacco smoke carcinogens, 15–25% of patients develop lung cancer without a significant personal history of tobacco use [1–3]. Lung cancer in never smokers (LCNS) (less than 100 cigarettes in their lifetime) is becoming a growing health problem and is now recognized as the seventh cause of cancer-related mortality worldwide [4]. Significant gender, geographical, histo-pathological, molecular and clinical differences are recognized in patients who never smoked when compared to smokers with lung cancer [3]. LCNS occurs more frequently in women and adenocarcinoma histology predominates. Its incidence is higher in certain geographical regions including Asia where the lung cancer incidence in never smoking Asian women is 3–4 fold higher when compared to the age-adjusted non-smoking female population of Western countries [5, 6]. Certain genomic changes such as mutations of the epidermal growth factor receptor *(EGFR),* human epidermal growth factor 2 *(ERBB2)*, or v-Raf murine sarcoma viral oncogene homolog B gene *(BRAF)*, rearrangements of the anaplastic lymphoma kinase (*ALK*) gene, *ROS1* proto-oncogene receptor tyrosine kinase (*ROS1*), or ret proto-oncogene (*RET)* are found in tumors of LCNS [7–10].

A deeper understanding of the biology of LCNS may improve treatment and screening options for those patients. Comprehensive analysis from the ethnically divert, single cohort on demographics, clinical, molecular, pathological characteristics, treatment and survival of never smokers with lung cancer is missing from the literature.

The aim of our study was to perform a comprehensive analysis of demographic, clinical, pathological, molecular, treatment and survival data in LCNS diagnosed and/or treated at a comprehensive North American cancer centre, the University Health Network-Princess Margaret (UHN-PM) Cancer Centre.

## RESULTS

### Patients characteristics

We identified 712 consecutive LCNS patients, diagnosed and/or treated at the UHN-PM Cancer Centre from June, 1988 to January, 2015. Patient and tumor characteristics are presented in Table 1. Most patients were female (72%) with a median age at diagnosis of 62.2 years (18–94). Most were Caucasian (46%) followed by East Asian (42%), black (5%), South Asian (4%) and other ethnicities (3%). Environmental tobacco exposure (ETS) was documented in 16% of patient records and exposure to other recognized lung cancer risks factors in 8%. However, most patients (76%) did not have documented identifiable risk factor. (Table 1). The family history of lung cancer was documented in only 6% of patients’ records.

**Table 1:** Demographic, clinical, and molecular baseline patient characteristics

Demographics				
		Ethnicity		
Characteristic	All patients *N* = 712 (%)	Caucasian *N* = 327 (%)	Asian *N* = 300 (%)	Other *N* = 85 (%)
Age (years)				
Median	62.2	63.3	61.6	57.3
Range	18–94	(30–94)	(28–90)	(18–89)
Gender				
Male	201 (28)	89 (27)	77 (26)	35 (41)
Female	511 (72)	238 (73)	223 (74)	50 (59)
ECOG PS (patients with stage IV) (*N* = 549)	(*N* = 549)	(*N* = 246)	(*N* = 231)	(*N* = 72)
0	208 (37.9)	90 (37)	92 (40)	26 (36.1)
1	285 (51.9)	128 (52)	117 (50.6)	40 (55.6)
2	35 (6.4)	18 (7)	14 (6)	3 (4.2)
3	20 (3.6)	10 (4)	7 (3)	3 (4.2)
4	1 (0.2)	–	1 (0.4)	–
Exposure to known single risk factor for lung cancer				
Environmental tobacco exposure (ETS)	107 (15.0)	52 (15.9)	46 (15.3)	9 (10.6)
Industrial dust	12 (1.7)	3 (0.9)	7 (2.3)	2 (2.4)
Asbestos	8 (1.1)	6 (1.8)	1 (0.3)	1 (1.2)
Occupational exposure to radioactive substances	4 (0.6)	1 (0.3)	2 (0.7)	1 (1.2)
Patients with breast cancer treated with chest radiation	17 (2.4)	8 (2.4)	8 (2.7)	1 (1.2)
Cooking fumes	4 (0.6)	–	4 (1.3)	–
HPV infection	3 (0.4)	1 (0.3)	–	2 (2.4)
History of pulmonary tuberculosis/bronchiectasis	3 (0.4)	–	2 (0.7)	1 (1.2)
Multiple (ETS and history of breast cancer and chest radiation)	4 (0.6)	4 (1.2)	–	–
Other (EBV infection *N* = 1; HIV infection *N* = 2)	3 (0.4)	1 (0.3)	1 (0.3)	1 (1.2)
Unknown/not documented	547 (76.8)	251 (76.8)	229 (76.3)	67 (78.8)
Previous non-lung cancers				
All	120 (17)	69 (21)	44 (15)	7 (8)
One prior	107 (15)	59 (18)	42 (14)	6 (7)
Multiple prior	13 (2)	10 (3)	2 (0.7)	1 (1.2)
Family history of lung cancer				
Yes	41 (6)	10 (3)	27 (9)	4 (5)
Unknown/not documented	671 (94)	317 (97)	273 (91)	81 (95)
**Clinical characteristics**				
Clinical stage at diagnosis				
IA	108 (15)	65 (19.9)	31 (10.3)	12 (14.1)
IB	56 (8)	28 (8.6)	23 (7.7)	5 (5.9)
IIA	25 (3)	10 (3.1)	14 (4.7)	1 (1.2)
IIB	18 (3)	8 (2.4)	8 (2.7)	2 (2.3)
IIIA	56 (8)	20 (6.1)	32 (10.7)	4 (4.7)
IIIB	30 (4)	14 (4.3)	16 (5.3)	–
IV	419 (59)	182 (55.8)	176 (58.7)	61 (71.8)
Histopathology				
Adenocarcinoma	621 (87)	295 (90.2)	255 (85.4)	71 (83.5)
Squamous Cell	29 (4.1)	14 (4.3)	11 (3.7)	4 (4.7)
Large Cell	17 (2.4)	5 (1.5)	10 (3.3)	2 (2.4)
Adenosquamous	14 (2)	3 (0.9)	8 (2.7)	3 (3.5)
Small Cell	5 (0.7)	2 (0.6)	2 (0.7)	1 (1.2)
Other *(NSCLC-NOS, Mixed tumor, Carcinoid, Lymphoepithelioma*	24 (3.4)	8 (2.4)	12 (4)	4 (4.7)
First-line treatment for metastatic disease				
Systemic chemotherapy (platinum doublet)	197 (33.9)	91 (35.0)	79 (32.0)	27 (36.1)
Targeted treatment	190 (29.1)	59 (22.4)	89 (34.6)	42 (34.7)
Radiotherapy	47 (8.6)	25 (10.2)	19 (8.2)	3 (4.2)
Clinical trial	39 (7.1)	20 (8.1)	8 (3.5)	11 (15.3)
Observation	38 (6.9)	26 (10.6)	8 (3.5)	4 (5.8)
Systemic chemotherapy (single agent)	19 (3.3)	6 (2.4)	10 (4.3)	2 (1.4)
EGFR TKI (Unknown mutation status or *EGFR WT*)	17 (3.1)	7 (2.8)	10 (4.3)	–
Surgery	22 (4.0)	9 (3.7)	12 (5.2)	1 (1.4)
Chemo-radiotherapy	10 (2.0)	6 (2.4)	4 (2.2)	–
Unknown	11 (2.0)	6 (2.4)	5 (2.2)	–
Number of systemic therapy lines for metastatic disease in entire cohort with stage IV disease (at diagnosis and during follow–up				
0–1	252 (47)	116 (49)	107 (46.6)	29 (40.3)
2	115 (21)	43 (17.8)	54 (23.1)	18 (25)
3	87 (16)	48 (19.4)	28 (12.4)	11 (15.3)
>3	79 (14)	28 (11.3)	37 (15.8)	14 (19.4)
Unknown	16 (2)	11 (2.4)	5 (2.1)	–
**Molecular characteristics**				
Patients with metastatic disease and “druggable” tumor driver mutation (*N* = 269)				
*EGFR*	226 (84.0)	78 (80.4)	112 (85.5)	36 (87.8)
*ALK*	36 (13.4)	16 (16.5)	16 (12.2)	4 (9.8)
*ERBB2*	5 (1.6)	1 (1.03)	3 (2.3)	1 (2.4)
*BRAF*	2 (0.7)	2 (2.1)	–	–
Patients with metastatic disease treated with targeted treatment (*N* = 235)				
* EGFR*	202 (86)	67 (81.7)	103 (88.8)	32 (86.5)
* ALK*	30 (13)	14 (17.1)	12 (10.3)	4 (10.8)
* ERBB2*	3 (1)	1 (1.2)	1 (0.8)	1 (2.7)
Patients with brain metastases at diagnosis by mutation status (*N* = 96)				
EGFR	64 (67)	23 (63.9)	30 (66.7)	11 (73.3)
* EGFR exon 19 deletion*	37 (58)^*^	13 (56.5)	17 (56.7)	7 (63.6)
* EGFR exon 21 insertion*	27 (42)^*^	10 (43.5)	13 (43.3)	4 (36.4)
*EGFR* other	0 (0)^*^	0 (0)	–	–
*ALK*	10 (10.4)	4 (11.1)	5 (11.1)	1 (6.7)
Other	3 (3.1)	1 (2.8)	2 (4.4)	–
None^**^	17 (17.7)	6 (16.7)	8 (17.8)	3 (20)
Unknown	2 (2.1)	2 (5.6)	–	–
Patients with brain metastases at diagnosis and during follow–up by mutation status (*N* = 216)				
*EGFR*	103 (47.7)	38 (38.8)	48 (53.9)	17 (58.6)
*ALK*	16 (7.4)	7 (7.1)	8 (9.0)	1 (3.4)
Other	8 (3.7)	5 (5.1)	3 (3.4)	–
None^***^	31 (14.3)	16 (16.3)	11 (13.4)	4 (13.8)
Unknown	58 (26.9)	32 (32.6)	19 (21.3)	7 (24.1)

NSCLC-NOS: NSCLC not other specified

^*^Percentage value only for *EGFR*-mutant cohort (*N* = 64)

^**^5 tumors tested only for *EGFR*

10 tumors tested only for *EGFR* and *ALK*

2 tumors tested using multigene Next Generation Sequencing (NGS) assays

^***^7 tumors tested only for *EGFR*

17 tumors tested only for *EGFR* and *ALK*

1 tumor tested only for *EGFR, ALK* and *ROS-1*

8 tumors tested using multigene NGS assays

A history of prior, non-lung malignancy was present in 17% of patients; 15% had a single prior malignancy and 2% had multiple cancers (Table 1 and Supplementary Table 1). The most common single malignancy was breast cancer (33%) followed by thyroid cancer (11%), lower gastrointestinal malignancies (11%) and non-Hodgkin lymphoma (6%). Thyroid cancer was the most common malignancy (46%) in patients with multiple non-lung primary malignancies, followed by breast cancer (23%).

Most patients (87%) had lung adenocarcinoma (Table 1). The majority of patients (59%) presented with metastatic disease, 29% with operable stage I or II, and only 12% with locally advanced stage III (Table 1). Brain metastases were present in 23% of patients who presented with stage IV disease at diagnosis and the majority of these patients (67%) had *EGFR* mutations (Table 1). Most patients with metastatic disease at diagnosis (90%) had good performance status (ECOG 0–1) (Table 1).

### Mutation status

**Figure 1:**
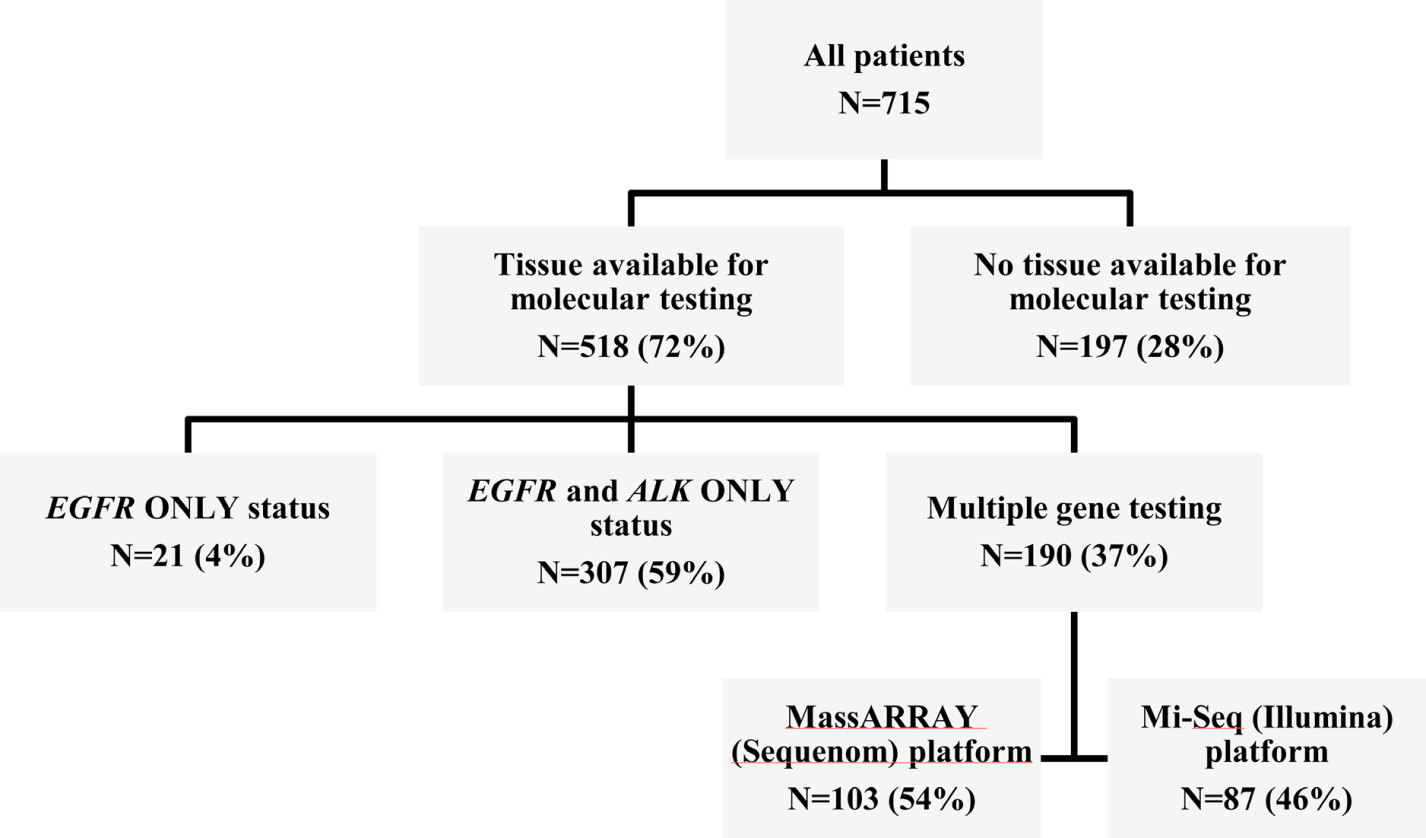
Summary of tumor tissue availability for genomic profiling

**Figure 2:**
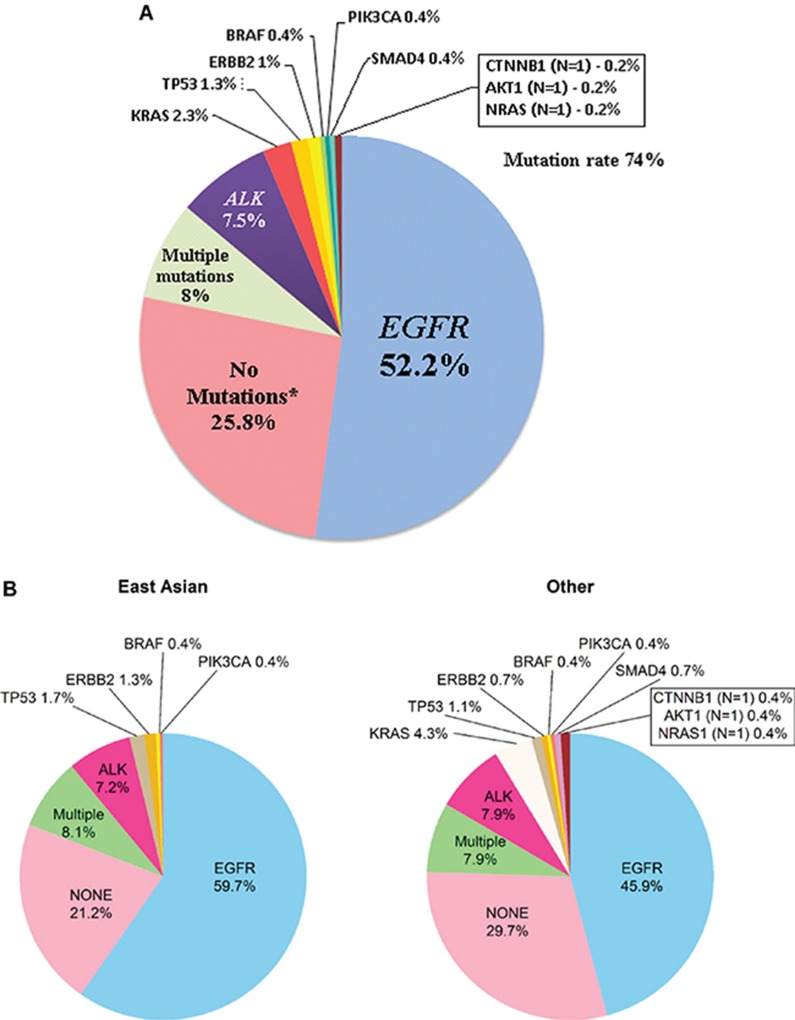
(**A**) Frequency of mutations (*N* = 518) in never smokers with lung cancer. ^*^21 tumor samples of patients were tested only for *EGFR*; 69 tumor samples were tested only for *EGFR* and *ALK* (1 out of 69 was tested also for *ROS-1*); 43 patients’ tumor samples were tested using multigene Next Generation Sequencing (NGS) assays: Sequenom MassARRAY (*N* = 22) and MiSeq Illumina (*N* = 21); 15 out of 43 available patients’ tumor samples with no detected mutations when tested with NGS assays were tested for *ROS-1*. (**B**) Frequency of mutations based on ethnicity. There are more Asian having EGFR: of the 236 Asian 155 had EGFR (66%); of the rest (*n* = 279), 141 had EGFR (51%) (Fisher exact *p* = 0.00066).

Out of 712 patients, 515 (72%) had tumor tissue available for molecular testing; 37% (188/515) of tumors were analyzed using NGS methods, while 59% (306/515) were tested only for *EGFR* and *ALK* (Figure 1). At least one mutation was present in 74% of analyzed tumors (Figure 2). Among 341 tumors with single mutations, *EGFR* (exon 19 deletions - 59.5%, exon 21 L858R point insertions - 38.7%) was the most common (78.9%), followed by *ALK* (11.4%), *KRAS* (3.5%), *TP53* (2.0%), *ERBB2* (1.5%), *BRAF* (0.6%), *PIK3CA* (0.6%), *SMAD4* (0.6%), *CTNNB1* (0.3%), *AKT1* (0.3%) and *NRAS* (0.3%) (Supplementary Table 2). Multiple mutations were present in only 8.0% of tumors (Figure 2, Supplementary Tables 2–4). Of 41 tumors with multiple mutations, 65.9% had *EGFR* as a co-mutation (Supplementary Table 3). Mutations were not detected in 25.8% of tumors (pan-negative) (Figure 2). None of pan-negative adenocarcinoma tumors available for further testing (*N* = 15) was positive for *ROS1*. The majority of *KRAS* mutations (single and multiple) were present in codon 12–81% (14/16), exclusively in adenocarcinoma tumors (16/16) and almost exclusively in Caucasians (12/16) (Supplementary Table 4).

### Treatment

Most patients with early stage disease (stage I-IIIA) underwent surgical resection (88%). Adjuvant cisplatin-based chemotherapy was delivered in all patients who had metastatic nodal involvement (31%). Stage IV patients (both at diagnosis and during the follow-up) with *EGFR* mutation, *ALK* translocation and *ERBB2* mutation positive tumors received targeted treatment in 88%, 83% and 67% cases, respectively.

The majority of patients with stage IV disease (83%) received first-line systemic (targeted or chemotherapy) treatment. In chemotherapy-treated patients (including patients who received chemo-radiotherapy) most received platinum-containing regimens (87%). Some patients (*N* = 49) with “druggable” driver mutation tumors received platinum-based systemic chemotherapy as first-line treatment (because of unknown tumor mutation status at the time of treatment decision or due to Provincial Guidelines) but subsequently received targeted treatment.

### Survival

**Figure 3:**
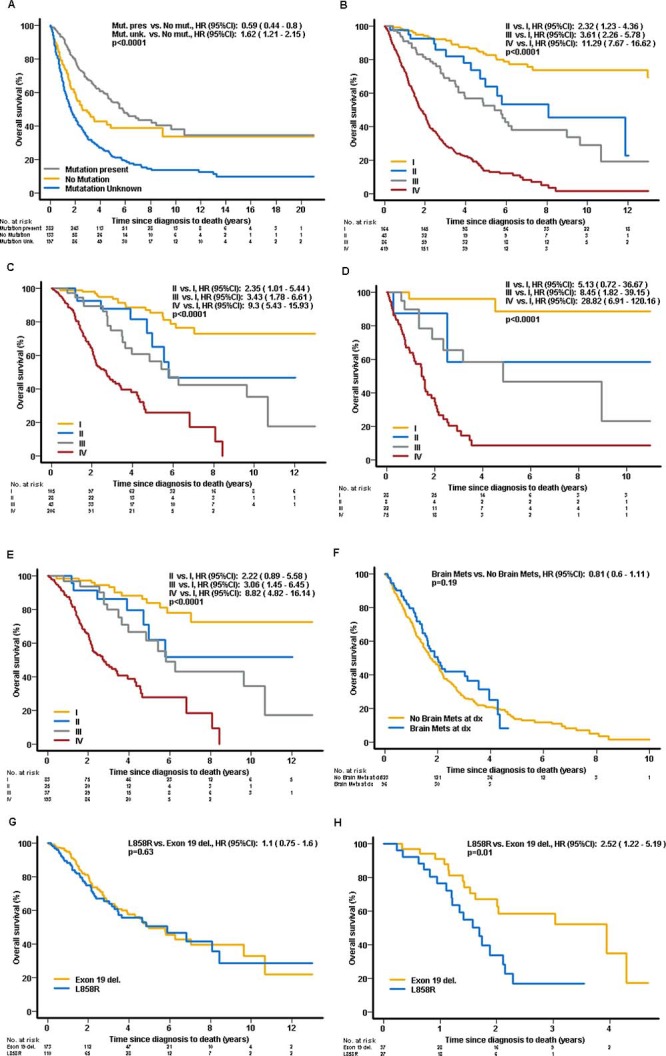
Overall survival (**A**) Entire cohort by mutation status at diagnosis. (**B**) Entire cohort by clinical stage at diagnosis (TNM staging). (**C**) All patients with mutation(s) by clinical stage at diagnosis (TNM staging). (**D**) All patients without mutations by clinical stage at diagnosis (TNM staging). (**E**) Patients with EGFR and ALK tumors by Clinical stage at diagnosis (TNM staging). (**F**) Patients with stage IV at diagnosis by presence of brain metastases at diagnosis. (**G**) Patients with EGFR-mutant positive tumors by the type of mutation – exon 19 deletion vs. L858R exon 21 insertion. (**H**) Patients with EGFR-mutant tumors and brain metastases at diagnosis by the type of mutation – exon 19 deletion vs. L858R exon 21 insertion.

The mOS for the entire cohort was 42.2 months (mo). Patients with mutations in their tumors had significantly longer OS when compared to those without (69.5 vs. 31.0 mo, respectively (hazard ratio [HR] 0.59; 95% confidence interval [CI] 0.44–0.79; *p <* 0.001). Patients with unknown tumor mutation status had the shortest survival (median 20.0 mo; HR 1.62; 95% CI: 1.21–2.16; *p* = 0.001) (Figure 3A). Early TNM stage (*p <* 0.001) (in the entire cohort – Figure 3B; in all patients with tumors with mutation(s) – Figure 3C; without mutations – Figure 3D; in all patients with *EGFR*-mutant and *ALK*-rearranged tumors – Figure 3E), adenocarcinoma histology (*p* = 0.012), good performance status (PS) (Eastern Cooperative Oncology Group, ECOG 0 vs. 1 vs. 2/3) at diagnosis (*p <* 0.001) and treatment with targeted therapy (*p <* 0.001) were each associated with longer OS. Patients with *ALK*-translocation-positive tumours have significantly longer OS compared to those without any mutations (HR = 0.33; 95% CI: 0.16–0.68; *p* = 0.0029) and to those with other and null mutations (HR = 0.44; 95% CI: 0.22–0.89; *p* = 0.022). Among the covariates included in the multivariable model (age, gender, stage, histology, ECOG and ethnicity) only gender, stage and ECOG PS were found significant. The significance was preserved for stage and PS when the model was performed for only the subset for which the mutation status was known (Supplementary Table 5). Gender (non-significant trends in OS) was retained in all final models.

Patients with *EGFR* mutated tumors had significantly shorter OS compared to the *ALK* cohort (mOS of 65 mo vs not reached, HR 2.16; 95% CI: 1.05–4.44; *p* = 0.036). In patients with stage IV disease at diagnosis, there was no significant difference in OS between patients with and without brain metastases at diagnosis (median 24.1 vs. 20.9 mo, respectively, HR 0.81, 95% CI: 0.6–1.11; *p* = 0.19) (Figure 3F). Among patients with *EGFR*-mutant tumors, there were no significant differences between patients with tumors carrying exon 19 deletions compared to patients harboring exon 21 insertions (median 59.6 vs. 70.4 mo, respectively, HR 1.1; 95% CI: 0.75–1.6; *p* = 0.64) (Figure 3G). In contrast, among patients with *EGFR*-mutant tumors with brain metastases *at diagnosis*, those with tumors harboring exon 21 L858R point insertions had significantly worse OS, when compared to those harboring exon 19 deletions (median 20.3 vs. 47.3 mo, respectively, HR 2.52; 95% CI: 1.22–5.19; *p* = 0.012) (Figure 3H). There was no significant differences based on ethnicity: Caucasian vs. Asian (median 50.5 vs. 40.8 mo; HR 0.95 95% CI: 0.76–1.19 *p* = 0.66); Caucasian vs. other ethnicities (median 50.5 vs. 38.3 mo; HR 0.91 95% CI: 0.74–1.12 *p* = 0.36) and Asian vs. other ethnicities (median 40.8 vs. 43.8 mo; HR 1.0 95% CI: 0.81–1.23 *p* = 0.99). Both South Asian and black ethnicity patients had the poorest outcome when compared with Caucasians or East Asians with a mOS of only 27 mo (compared with Caucasian: HR 1.48, 95% CI: 1.06–2.08, *p* = 0.002, and East Asian: HR 1.42, 95% CI: 1.01–2.01 *p* = 0.04).

Patients with “druggable” driver mutation tumors who received platinum-based systemic chemotherapy as first-line treatment but subsequently received targeted treatment had similar survival when compared to patients who received targeted therapy as the first line (36% vs. 33% 5-year survival; *p* = 0.71).

## DISCUSSION

With 712 patients, our study is the largest series of LCNS diagnosed and/or treated at a single institution that includes comprehensive demographic, clinical and molecular features of LCNS. We demonstrate that despite the high proportion of patients presenting with stage IV cancers, and even brain metastases, LCNS is associated with prolonged OS, most likely due to the high prevalence of actionable driver mutations.

LCNS now accounts for ~25% of all lung cancer cases worldwide [3, 6, 11]. Whether the incidence of LCNS is rising in the global population or there is simply an increase in the ratio of never smokers to ex- or current smokers among patients diagnosed with lung cancer remains a subject of ongoing debate [5, 12, 13]. In Pacific Rim countries LCNS accounts for 30–40% of all patients diagnosed with lung cancer, and in Taiwan and Korea the proportion of patients diagnosed with lung cancer with no history of tobacco consumption (mostly women) can be as high as 75–90% compared to ~15% in North America and Europe [5, 14]. In a single institution studies from South Asia (India) the proportion of never smokers with lung cancer varied between 31–52% with most (88–94%) being females [15].

LCNS has been recognized only recently as a distinct clinical entity and has become the subject of intensive basic and clinical research [3, 6, 16]. The high prevalence of “druggable”/actionable driver gene mutations in LCNS tumors, geographical and ethnic differences in its incidence and clinico-pathological and genomic features, make LCNS a unique disease among thoracic malignancies [6, 17].

In our cohort, which is similar to previously published reports, women represented the majority (72%); most tumors (88%) were adenocarcinomas and the 59% of patients had metastatic disease at diagnosis [3, 18]. Historically, the majority of studies and reports have come from East Asia due to the high incidence of LCNS in this geographic region, with only few recently published reports from Western countries [19–21]. Caucasians and East Asians constituted the most common ethnicities (46% and 42%, respectively) in our cohort. The PM Cancer Centre's unique location in one of the most multi-cultural and multi-ethnic cities in the world, allowed us to study LCNS among ethnically diverse populations including Caucasian, East Asian, South Asian, and black patients. Median age of diagnosis in our study was 62.2 years for entire cohort with no gender differences: 62.4 (women) and 61.6 (men) years, respectively. While reports from East Asia indicate the higher incidence of LCNS at younger ages, this finding was found in some but not all cohort analyses of Western populations [6, 12, 22, 23]. In our study, there was no significant difference in age and gender distribution at diagnosis between the Caucasian and Asian populations (Table 1). There was, however, a significantly (*p* = 0.0028) smaller proportion of stage I disease at diagnosis among the Asian population (18%) as compared to the Caucasian population (28%) (Table 1).

There are several recognized risk factors besides tobacco smoking in the pathogenesis of lung cancer; in LCNS environmental tobacco exposure is the most established of these risk factors [24, 25]. Other putative risk factors include exposure to radon, domestic (cooking) fumes (especially in East Asia), asbestos, air pollution, hormonal factors, presence of pre-existing lung disease, previous treatment with ionizing radiation to the chest, oncogenic viruses (e.g. human papillomavirus – HPV) or inherited genetic susceptibility [26–28]. In our retrospective study, only 16% of patients had documented environmental tobacco exposure in their medical records. According to the most recent, prospective epidemiological study on LCNS from France, definite exposure to the most common occupational carcinogens can be as high as 35% in men but only 8% in women [21]. The retrospective nature of our study, however, limits the accuracy of the reported potential carcinogen exposure in our studied population.

Approximately 45% of men and 38% of women will be diagnosed with some form of invasive cancer during their lifetime; 8–10% of all newly diagnosed cancers will occur in patients with a prior diagnosis of a different malignancy [29]. The relatively high (16%) incidence of prior non-lung primary malignancies in our cohort has not been reported previously. The occurrence of multiple cancers in an individual could be partially explained by inherited genetic mutations conferring susceptibility to the relevant cancers, and partially due to environmental exposures increasing the risk of multiple cancers or long term toxicities of therapy used for the first cancer [30]. Our finding however requires further validation.

The most common mutation in lung adenocarcinoma in never smokers is *EGFR* and its incidence varies depending on gender and ethnicity being the highest in East Asian females (~78%), followed by Caucasians (43–51%) and South Asians (29%) [19, 21, 31, 32]. In concordance to recent reports from Europe and Asia, in our study *EGFR* was the most common single mutation (52.1%) and exon 19 deletions were the most common subtype (60%) of *EGFR* followed by exon 21 insertions (38.5%) [20, 21].

Patients harboring *EGFR* exon 19 deletions compared to those harboring exon 21 insertions may have longer survival [33, 34]. In our study we found no *significant* survival difference between these two patients’ cohorts (Figure 3G). However, in patients with brain metastases and *EGFR*-mutant tumors, the presence of exon 21 insertion was associated with significantly shorter survival (HR 2.52; 95% CI: 1.22–5.19; *p* = 0.01) when compared to patients with tumours harbouring exon 19 deletion. The reason for this difference remains largely unknown and may be only partially explained by higher affinity of EGFR tyrosine kinase inhibitors (EGFR-TKIs) to EGFR with exon 19 deletion compared to exon 21 insertion, combined with drug exposure variability across the blood-brain barrier [35, 36].

Presence of brain metastases in unselected NSCLC patients historically confers worse prognosis with less than 50% of patients surviving longer than six months [37]. Brain metastases were present in 23% of patients with stage IV disease at presentation and 67% had *EGFR*-mutant tumors. Patients with brain metastases at diagnosis and with tumours harboring mutations had significantly better OS when compared to those with brain metastases and those harboring no identifiable mutations – mOS of 26 mo vs. 17 mo, respectively (HR = 0.33, 95% CI 0.17–0.64; *p <* 0.001). There was no significant difference in mOS between patients with and without brain metastases at diagnosis – 24.1 vs. 20.9 mo, respectively (HR = 0.81, 95% CI 0.60–1.11; *p* = 0.19). Among patients with brain metastases at diagnosis, ~80% of patients had tumors with two most common “druggable” mutations (67% - *EGFR*, 10% - *ALK*) with known high response rates of CNS disease to targeted TKIs and evidence of higher response rates to WBRT when compared to wild type tumors; this may explain the lack of survival difference between patients with and without brain metastases in our cohort [38–42].

Before the era of molecularly targeted therapy, many but not all retrospective analyses, showed better survival of LCNS when compared to smokers [18, 43–45] that was independent of other known prognostic factors [46, 47]. The better outcome of LCNS may be explained in part by the predominance of women and adenocarcinoma histology since these two factors have long been recognized as favorable prognostic factors in NSCLC [48]. However, the identification of sensitizing driver mutations in the non-smoking population provided the unique opportunity for molecularly selected lung cancer patients to receive targeted, personalized treatment that translate into clinically meaningful benefit compared to patients with tumors lacking actionable genomic drivers [49, 50]. In our study, we report a mOS of 42.2 mo for the entire cohort, with significantly better outcome for patients with tumors harboring mutations (69.5 mo) when compared to patients without mutations (31.0 mo) or with unknown mutation status (19.9 mo). Our results are similar to those recently published by Kris *et al*. [50]

Our study has limitations since it is a retrospective analysis of highly selected patients diagnosed and treated from 1988–2015 in a tertiary referral academic hospital. During that time, we have witnessed rapid translation of basic discoveries into practice changing treatment guidelines and worldwide implementation of molecular testing in NSCLC patients. We are aware about heterogeneity of genetic testing (moving from single gene mutation/translocation analysis to multiplex NGS), treatment modalities (incorporation of targeted agents in the routine practice for selected patients) and sequence of these therapies in our cohort, which reflects rapidly evolving diagnostic and treatment guidelines for patients with NSCLC in the recent years.

In summary, we report on epidemiological, clinical, pathological, molecular and survival data of the largest cohort of never smokers with lung cancer from a single institution. We demonstrated that LCNS is characterized by prolonged survival, particularly in the presence of actionable driver mutations that allow personalized treatment options that translate into clinically meaningful benefit.

## PATIENTS AND METHODS

### Patients

We performed a retrospective analysis of demographic, clinical and laboratory data stored in the electronic patient record system at the UHN-PM Cancer Centre of never smokers with a pathologic diagnosis of primary lung malignancy diagnosed and/or treated from June 1988 to January 2015. The study was approved by the institutional Research Ethics Board (REB).

### Data collection

We collected the following data: gender, ethnicity, age at diagnosis, weight and height, performance status at diagnosis, TNM stage at diagnosis, histopathology, molecular pathology data (somatic tumor mutations), type and duration of systemic treatments and survival history. When available in patients’ history, we collected the data on exposure to potential carcinogens, family history of lung cancer, history of non-lung primary malignancies and types of received treatment(s).

### Molecular testing

Molecular testing was performed using paraffin-embedded archival tumor tissue (Supplementary data 1). Since March 2010, routine testing for *EGFR* (exon 19 deletions and exon 21 insertions) and *ALK* is performed on all locally advanced/metastatic non-squamous NSCLC tumor specimens in the Molecular Diagnostics Laboratory at the UHN. At the time of final analysis *EGFR* testing for exons 18, 19 and 20 was not routinely performed. Among 712 patients, 515 (72%) patients had molecular data available in the medical records and/or had adequate tissue available for testing to obtain the data on molecular abnormalities (Figure 1). Due to low likelihood of co-existing driver mutations, no further gene testing was performed on tumor samples harbouring *EGFR* mutations or *ALK* translocations on routine testing. *EGFR* wild-type and *ALK*-rearrangement negative (*EGFR-WT/ALK-WT*) tumors were tested further using MassARRAY technology (Sequenom, San Diego, CA) or MiSeq (Illumina, San Diego, CA, USA) next-generation sequencing (NGS) personal genomics platforms (Supplementary Data 1), when adequate archival tumor tissue was available (Figure 1). NGS analysis was performed in a UHN laboratory certified by the College of American Pathologists and Certified Laboratory Improvements Amendments. “Pan-negative” (by NGS) adenocarcinoma tumors from patients who were alive at the time of final analysis and have the tumour core biopsy or surgical specimen available (*N* = 15) were tested for *ROS1* rearrangements using *ROS1* break-apart probe set using a paraffin pretreatment reagent kit (Vysis, Abbott Laboratories, Abbott Park, IL, USA (Supplementary Data 1).

### Statistical methods

The main outcome was overall survival (OS) calculated from the diagnosis date to the date of death or last follow-up visit. Median OS (mOS) was determined using Kaplan-Meier estimates and *p*-values expressing the difference between the survivor distributions were based on the Wald test within the Cox proportional hazards model. When covariates had more than two levels the overall *p*-value was based on the log-rank test. The effect of *EGFR* and *ALK* molecular alterations also was tested while adjusting the model for significant clinical factors. The covariates of age, gender, stage, histology (adenocarcinoma vs. all other histologies), ECOG and ethnicity were included in the model. Utilizing a backward stepwise selection method, covariates that were not significant were excluded one by one from the model. The covariates thus selected were also tested in the subset for which the mutation status was known. The *EGFR* mutations and *ALK* translocations were tested adjusting the model for the significant covariates found in the previous step.

## SUPPLEMENTARY MATERIALS TABLES


