# Supplementary material for: Lung cancer in never smokers from the Princess Margaret Cancer Centre
Source: Oncotarget. 2018 Apr 27;9(32):22559–70. doi: 10.18632/oncotarget.25176 (PMC5978248; doi:10.18632/oncotarget.25176)
Supplement: Supplementary file 1 [file oncotarget-09-22559-s001.pdf]

Lung cancer in never smokers from the Princess Margaret Cancer Centre

SUPPLEMENTARY MATERIALS

ALK testing

For *ALK* rearrangements, tumor samples were assessed first by immunohistochemistry using anti-ALK (CD246) (p80) (clone 5A4) mouse monoclonal antibody (Leica Biosystems) according to manufacturer's protocol. Confirmatory fluorescence in-situ hybridization (FISH) analysis using the ALK break-apart probe (Abbott Molecular, Chicago, IL) was performed on tumor samples with positive staining by immunohistochemistry [1–3].

ALK immunohistochemistry

Immunohistochemistry for ALK was performed on 4 μm -thick formalin-fixed, paraffin embedded tissue sections using clone 5A4 (Leica Biosystems). Briefly, slides were deparaffinized, then treated with Peroxidase Block (DAKO, Carpinteria, CA) for 15 minutes to quench endogenous peroxidase activity. Antigen retrieval was carried out in citrate buffer (pH 6) in a pressure cooker at 122° C for 30–45 minutes. The sections were then incubated with the primary mouse monoclonal anti-ALK antibody at a 1:50 dilution for 40 minutes, washed in 50 mM Tris-HCl (pH 7.4), and incubated with horseradish peroxidase–conjugated secondary antibodies (Envision Plus detection kit, DAKO). Staining was developed through incubation with diaminobenzidine (DAB), and sections were counterstained. The stained slides were reviewed by a pathologist and staining results were graded semiquantitatively as follows: 0 for absent or barely perceptible expression in rare cells, 1 (low) for weak to moderate multifocal expression and 2 (high) for strong staining in most cells. All positive cases demonstrated a granular, cytoplasmic expression pattern. Focal, weak rimming of intracellular mucin droplets was considered negative.

ALK fluorescence *in situ* hybridization (FISH) staining protocol

FISH was performed on unstained paraffin sections of standard thickness (4 um) on charged (coated) slides. Briefly, unstained slides were baked at 55° C, de-paraffinized in xylene, dehydrated in ethanol, and air-dried. Slides were then incubated in citrate buffer at 80° C, followed by pepsin digestion at 37° C, and dehydration in ethanol. Slides and probe were codenatured for 5 min at 74° C and hybridized overnight at 37° C. After washing in saline sodium citrate (SSC)/NP-40 solution, slides were counterstained with 4′,6-diamidino-2-phenylindole, dihydrochloride (DAPI) mixed with mounting medium.

EGFR testing

Genomic DNA was extracted from macro-dissected unstained paraffin-embedded sections (5 per sample, 5 μm sections), based on a representative H&E stained slide with the tumor area for macrodissection being circled for histology samples or the same number and thickness of complete sections for cytology cell blocks. DNA quality and quantity was assessed by a spectrophotometer and the quality of DNA was also assessed using agarose mini gel electrophoresis. Positive controls used were extracted cell-lines at 100% (1 copy), 5%, and 1%: HCC827 (#107) – exon 19 mutant using a 1:12 dilution due to high copy number of exon 19 mutant alleles in the cell line, and H3255 (#103) – exon 21 mutant. Negative control DNA (placenta or other *EGFR* mutation negative tissue) was used to dilute the positive control cell lines.

Detection of exon 19 deletions was done through fragment analysis following fluorescently-labelled polymerase chain reaction (PCR) using the following primers:

EGFR-Ex-19-FWD1: GCA CCA TCT CAC AAT TGC CAG

EGFR-Ex-19-REV1-FAM: 6FAM-AAA AGG TGG GCC TGA GGT TCA).

Master mix per reaction was created using 2.5 μL 10X PCR Buffer (Applied Biosystems ABI), 1.5 μL 25 mM $MgCl_2$, 0.2 μL 25 mM dNTP (mixture of equal amounts dATP, dCTP, dGTP, dTTP – Amersham #27203501), 0.2 μL EGFR-Ex-19-FWD1, 0.2 μL EGFR-Ex-19-REV1-FAM, 0.1 μL Ampli TaqGold (5 U/μL – ABI

P/N 10966-034), and 10.3 µL dH_2O to create a total of 15 µL per reaction.

Detection of L858R single base-pair substitution of exon 21 was done through PCR-RFLP (restriction fragment length polymorphism) following Sau96I (5 U/µL – New England BioLabs – Cat #R0165S) restriction enzyme digest targeting GGNCC. The following primers were used:

EGFR-Ex-21-FWD1: CCT CAC AGC AGG GTC TTC TCT GT

EGFR-Ex-21-REV1-FAM: 6FAM-TCA GGA AAA TGC TGG CTG ACC TA

Master mix per reaction was created using 2.5 µL 10X PCR Buffer, 1.5 µL 25 mM $MgCl_2$, 0.2 µL 25 mM dNTP, 0.2 µL EGFR-Ex-21-FWD1, 0.2 µL EGFR-Ex-21-REV1-FAM, 0.2 µL Ampli TaqGold, and 10.2 µL dH_2O to create a total of 15 µL per reaction.

Approximately 100 ng of patient DNA was diluted with H_2O to provide a total volume of 10 µL. For poor quality samples (highly degraded or very low concentration) a maximum volume of 5 µL of DNA and 5 µL H_2O was used. PCR set-up for both reactions was done using 15 µL of master mix together with 10 µL of [patient DNA + H_2O]. PCR conditions for both reactions were as follows: 95° C for 10 min, 35 cycles of [95° C for 30 seconds, 65° C for 30 seconds, 72° C for 45 seconds], 72° C for 5 minutes, and hold at 4° C. For exon 21 PCR restriction enzyme digest, a master mix per reaction was created using 2.0 µL 10X NEBuffer 4 (New England BioLabs B7004S), 0.5 µL Sau96I (5 U/µL), and 7.5 µL H_2O for a total of 10 µL per reaction. The master mix was then added to 10 µL of exon 21 PCR products and incubated at 37° C for 2.5 hours, then hold at 4° C.

Electrophoresis was performed on ABI 3130×l/3500 Genetic Analyzer using 1 µL of exon 19 PCR product and 2 µL of exon 21 digest product to wells containing HiDi formamide (P/N 4311320) and GeneScan-350 Rox standard mixture (P/N 401735). Using ABI 3130×l or 3500 Data Collection software, the following allele sizes were observed:

Exon *19*:	Wild type allele	207 bp
	Mutant allele	<207 bp
	Non-specific	194-195 bp
Exon 21:	Wild type allele	174-175 bp
	Mutant allele	86 bp
	Undigested	222 bp

ROS1 testing

ROS1 immunohistochemistry

Sections were deparaffinized and incubated with the rabbit primary monoclonal ROS1 antibody [4] (Clone D4D6, #3287, Cell Signaling Technology, Danvers, MA, USA) at a dilution of 1:50 2 h at 20° C. A standard three-stage indirect immunoperoxidase technique was performed on a Benchmark XT Ventana staining module using an XT UltraView DAB kit; antigen retrieving was performed with the Cell Conditioning buffer (CC1, Tris/Boric acid/EDTA pH8.0) for 1 h, according to the manufacturer's instructions (Ventana, Tucson, AZ, USA). No signal amplification was required. The specificity of the ROS1 antibody was assessed by using paraffin-embedded cell blocks of the ROS1-rearranged cell line HCC78, which was subsequently used as positive external controls for all tests. Normal lung tissues were used as negative controls. The percentage of positive cells was evaluated, and staining scores were assessed as follows: 0; no staining, 1+; faint cytoplasmic staining, 2+; moderate cytoplasmic staining and 3+; intense granular cytoplasmic staining, similarly to previously described [4]. Membrane staining was recorded when observed.

ROS1 fluorescence *in situ* hybridization (FISH)

FISH was performed on unstained 3–4 m formalin-fixed paraffin embedded (FFPE) tumor tissue sections with the use of a *ROS1* break-apart probe set using a paraffin pretreatment reagent kit (Vysis, Abbott Laboratories, Abbott Park, IL, USA), as described previously [5]. Commercially available *ROS1* break-apart FISH probe set was used for this study: the Aquarius Pathology *ROS1* Breakapart Probe (Cytocell, Cambridge, GB). The Aquarius Pathology probe set consists of a green 406 Kb 5 (telomeric) probe and two red (299 Kb and 171 Kb) 3 (centromeric) probes, positioned on each side of the *ROS1* gene. The probe was tested on the *ROS1*-rearranged patient sample. Nuclei were counterstained with DAPI/Vectashield (Vektor Laboratories, Burlingame, CA, USA) and were analyzed with a Metafer slide scanning system (Metasystems, Altlussheim, Germany) under a 63X oil immersion objective with a fluorescence microscope (Imager Z2, Zeiss) equipped with appropriate filters, a charge-coupled device camera, and the FISH imaging and capturing software Metafer 4 (Metasystems). Signals were enumerated with the ISIS imaging system (Metasystems). Tumor tissues were considered *ROS1* FISH positive (*ROS1*-rearranged) if >15% tumor cells showed split red and green signals (signals separated by ≥1 signal diameter) and/or single 3 signals. Otherwise the samples were considered as FISH negative.

Sequenom and Mi-Seq next generation sequencing technology for multiple gene testing

Genomic tumor DNA was extracted from available patient tumor biopsy tissue or surgical resection specimens and was analyzed for known somatic mutations using MassARRAY technology (Sequenom, San Diego, CA) or MiSeq (Illumina, San Diego, CA, USA) next-generation sequencing (NGS) personal genomics platform and verified by Sanger sequencing [6]. After macro-dissection, tissues were deparaffinized with xylene then treated with proteinase K treatment prior to DNA extraction. DNA was extracted in a College of American Pathologists (CAP) and Certified Laboratory Improvements Amendments (CLIA) certified laboratory using the QIAmicro DNA extraction kit (QIAGEN, Hilden, Germany), according to the manufacturer's instructions.

The MassArray assay used 10–20 ng of DNA and the Illumina TruSeq assay used 250 ng of DNA.. Molecular profiling was performed using a customized multiplex MassARRAY Sequenom panel including 23 genes (*AKT1,*

AKT2, AKT3, BRAF, CDK4, CTNNB1, EGFR, ERBB2, FGFR1, FGFR2, FGFR3, HRAS, KIT, KRAS, MEK1, MET, NOTCH1, RAS, PDGFRA, PIK3CA, RET, SMO, STK11) and 279 mutations on the next generation sequencing (NGS) Illumina MiSeq TruSeq Amplicon Cancer Panel including 48 genes (*ABL1, AKT1, ALK, APC, ATM, BRAF, CDH1, CSF1R, CTNNB1, EGFR, ERBB2, ERBB4, FBXW7, FGFR1, FGFR2, FGFR3, FLT3, GNA11, GNAQ, GNAS, HNF1A, HRAS, IDH1, JAK2, JAK3, KDR, KIT, KRAS, MET, MLH1, MPL, NOTCH1, NPM1, NRAS, PDGFRA, PIK3CA, PTEN, PTPN11, RB1, RET, SMAD4, SMARCB1, SMO, SRC, STK11, TP53, VHL*) with 212 amplicons and ≥500x coverage in our CLIA-certified laboratory. For the Sequenom MassArray assay, DNA was amplified using the OncoCarta PCR primer mix. Unincorporated nucleotides were inactivated by shrimp alkaline phosphatase (SAP). A single base extension reaction was performed using primers that hybridize immediately adjacent to the mutation. Q cation exchange resin was added to remove salts. Multiplexed reactions were spotted onto the SpectroChipII using the MassARRAY Nanodispenser. Peaks with different mass were resolved by matrix-assisted laser desorption/ionization time-of-flight on the MassARRAY Compact Analyzer. Data analysis was performed using MassARRAY Typer Analyzer 4.0.20 software which generates a report of specific mutations and ratio of mutant frequency. Mutant peaks were verified by manual review of all data.

Sample processing

Formalin-fixed paraffin-embedded tumor samples were macrodissected or cored, deparaffinized, treated with Proteinase K, and DNA extracted using the QIAamp DNA micro kit (QIAGEN), according to manufacturer's instructions. All DNA samples were quantitated by Qubit, and had their quality checked using Illumina's FFPE QC test kit.

Amplicon-based targeted sequencing

Targeted sequencing was carried out according to lab-standard protocols incorporating the following steps: hybridization of the adapter oligonucleotide pool to the samples; removal of unbound oligonucleotides; extension-ligation of bound oligonucleotides; PCR amplification and subsequent clean-up; library normalization; and, library pooling and loading of the MiSeq. The Illumina MiSeq utilizes bridge amplification, cluster generation and sequencing by synthesis to enable highly parallel DNA sequencing of multiple samples simultaneously. The incorporation of fluorescently labeled reversible terminator nucleotides was detected by laser excitation and imaging during each sequencing cycle.

Data analysis

Data analysis was accomplished using the NextGENe v 2.3.1 (SoftGenetics) software package. Briefly, FASTQ files for each sample were generated from the raw image data, aligned to build 37 of the human reference genome, and all nonsynonymous coding and splice site variants (including frameshift and indel variants) with > 5% allele frequency called. Synonymous variants, and those that are known polymorphisms in the 1000 genomes database, were excluded. Data were reviewed manually to ensure adequate coverage (> 500x) in regions where variants were called, and to ensure data quality.

REFERENCES

1. . Cutz JC, Craddock KJ, Torlakovic E, Brandao G, Carter RF, Bigras G, Deschenes J, Izevbaye I, Xu Z, Greer W, Yatabe Y, Ionescu D, Karsan A, et al. Canadian anaplastic lymphoma kinase study. A model for multicenter standarization and optimization of ALK testing in Lung Cancer. J Thorac Oncol. 2014; 9:1255–1263.
2. McLeer-Florin A, Moro-Sibilot D, Melis A, Salameire D, Lefebvre C, Ceccaldi F, de Fraipont F, Brambilla E, Lantuejoul S. Dual IHC and FISH testing for ALK gene rearrangement in lung adenocarcinomas in a routine practice: a French study. J Thorac Oncol. 2012; 7:348–354.
3. Sholl LM, Weremowicz S, Gray SW, Wong KK, Chirieac LR, Lindeman NI, Hornick JL. Combined Use of ALK immunohistochemistry and FISH for optimal detection of ALK- rearranged lung adenocarcinomas. J Thorac Oncol. 2013; 8:322–328.
4. Rimkunas VM, Crosby KE, Li D, Hu Y, Kelly ME, Gu TL, Mack JS, Silver MR, Zhou X, Haack H. Analysis of receptor tyrosine kinase ROS1-positive tumors in non-small cell lung cancer: identification of a FIG-ROS1 fusion. Clin Cancer Res. 2012; 18:4449–4457.
5. Acquaviva J, Wong R, Charest A. The multifaceted roles of the receptor tyrosine kinase ROS in development and cancer. Biochim Biophys Acta. 2009; 1795:37–52.
6. Collins FS and Hamburg MA. First FDA authorization for Next-Generation Sequencer. N Engl J Med. 2013. 369:2369–2371.

Supplementary Table 1: Prior non-lung malignancies (*N* = 120)

Cancer type – single (*N* = 107)	*N*
Breast	36
Thyroid	11
Colorectal/Anal	11
Non-Hodgkin Lymphoma	7
Head & Neck	5
Endometrial	5
Cervical	3
Gastric	3
Ovarian	3
Prostate	3
Meningioma	2
Soft Tissue Sarcoma (lower limb)	2
Other*	16
**Prior cancer types – multiple (*N* = 13)**	
Breast cancer, endometrial cancer	1
Breast cancer, colon cancer	1
Breast cancer, melanoma	1
Thyroid cancer, seminoma	1
Thyroid cancer, ALL, H&N, Basal skin carcinoma	1
Thyroid cancer, breast cancer	1
Thyroid cancer, multiple myeloma	1
Thyroid cancer, renal cancer	1
Thyroid cancer, rectal cancer, H&N	1
Colon cancer, cervical cancer	1
Prostate, renal cancer	1
Melanoma, Retinoblastoma	1
Pancreatic cancer, bladder cancer, renal cancer	1

*The individual cancers were: vulva squamous cell carcinoma, glioblastoma multiforme, peritoneal mesothelioma, myxoma peritonei, prolactinoma, pancreatic adenocarcinoma, seminoma, melanoma, chronic lymphocytic lymphoma, skin squamous cell carcinoma, carcinoid, hepatoma, mucoepidermoid lung tumor, nasopharyngeal cancer, nephroblastoma

Supplemantary Table 2: Mutation frequency in patients with detected single gene mutations (*N* = 341)

Mutation	*N* (%)
EGFR	269 (78.9)
ALK	39 (11.4)
KRAS	12 (3.5)
TP53	7 (2)
ERBB2	5 (1.5)
BRAF	2 (0.6)
PIK3CA	2 (0.6)
SMAD4	2 (0.6)
CTNNB1	1 (0.3)
AKT1	1 (0.3)
NRAS	1 (0.3)

Supplementary Table 3: Detailed characteristics of multiple mutations/translocations

	**Multiple mutations (*N* = 41)**	
1.	***ALK***; ***TP53*** *p.Glu285Lys*	
2.	***ALK; TP53*** *p.Ile332Asn*	***ALK*** **and other genes (*N* = 3)**
3.	***ALK***; ***KIT*** *p.Met541Leu;* ***TP53*** *p.Lys164Met*	
4.	***BRAF*** *p.Gly469Ala;* ***TP53*** *p.Tyr163Cys;* ***SMO*** *p.Cys550Phe*	***BRAF*** **and other genes (*N* = 1)**
5.	***EGFR*** *p. Glu709Ala;* ***EGFR*** *p. Gly719Ser*	
6.	***EGFR*** *p.Leu861Gln;* ***EGFR*** *p.Gly719Ser*	***EGFR*** **double mutations (*N* = 5)**
7.	***EGFR*** *p.Gly719Ala;* ***EGFR*** *p.Leu861Gln*	
8.	***EGFR*** *exon 19 del;* ***Exon*** *18 p.Gly719X*	
9.	***EGFR*** *p.Leu858Arg;* ***CTNNB1*** *p.Ser37Cys*	
10.	***EGFR*** *p.LeuL858Arg;* ***CTNNB1*** *p.Ser37Cys*	
11.	***EGFR*** *p.Ser768_Asp770dup;* ***CTNNB1*** *p.Asp32Gly*	
12.	***EGFR*** *p.Gly719Ser;* ***EGFR*** *p.Ser768Ile;* ***CTNNB1*** *p.Ser33Cys*	
13.	***EGFR*** *p.GluE746_Ala750del;* ***HER-4*** *p.Ser302Ile;* ***KIT*** *p. Ala736Thr*	
14.	***EGFR*** *p.His773_Val774dup exon 20;* ***IDH1*** *p.Arg132Cys*	
15.	***EGFR*** *p.Glu746_Ala750del;* ***PIK3CA*** *p.His1047Arg*	
16.	***EGFR*** *p.Leu858Arg;* ***PTEN*** *p.Gly230Ala*	
17.	***EGFR*** *p.Leu747_Thr751del;* ***PTEN*** *p.Glu242X*	
18.	***EGFR*** *p.LeuL747_Thr751del;* ***RB1*** *p.Lys745fs*	
19.	***EGFR*** *p.Leu858Arg;* ***SMAD4*** *p.Gln455X*	
20.	***EGFR*** *p.Leu747_Ser752del;* ***STK11*** *p.Phe354Leu*	***EGFR*** **and other genes (*N* = 22)**
21.	***EGFR*** *p.Glu746_Ala750del;* ***TP53*** *p.Cys135Tyr*	
22.	***EGFR*** *p.Leu747_Ser752del;* ***TP53*** *p.Ser241Phe*	
23.	***EGFR*** *p.Glu746_Ala750del;* ***TP53*** *p.Val274Phe*	
24.	***EGFR*** *p.Leu747Pro;* ***TP53*** *p.Gly245Val*	
25.	***EGFR*** *p.Leu858Arg;* ***TP53*** *p.Ala161Thr*	
26.	***EGFR*** *p.Glu746_Ala750del;* ***TP53*** *p.Tyr163Asn*	
27.	***EGFR*** *p.Glu746_Ala750del;* ***TP53*** *p.Met160fs*	
28.	***EGFR*** *p.Glu746_Ala750del;* ***TP53*** *p.Arg249Thr*	
29.	***EGFR*** *p.Ser752_Ile759del;* ***TP53 p.****Cys238Arg*	
30.	***EGFR*** *p.Ile740_Lys745dup;* ***TP53*** *p.Tyr205Cys*	
31.	***EGFR*** *p.LeuL858Arg;* ***TP53*** *p.His241Arg;* ***GNAQ*** *p.Val314Met*	
32.	***ERBB2*** *p.Ala775_Gly776ins;* ***TP53*** *p.Tyr234Cys*	***ERBB2*** **and other genes (*N* = 2)**
33.	***ERBB2*** *p.Tyr772_Ala775dup;* ***TP53*** *p.Gln136fs*	
34.	***KRAS*** *p.Gly12Val;* ***KIT*** *p.Ile744Thr*	
35.	***KRAS*** *p.Gly12Val;* ***TP53*** *p.Gln104X*	***KRAS*** **and other genes (*N* = 4)**
36.	***KRAS*** *p.Gly12Val;* ***MET*** *p.Asn375Ser*	
37.	***KRAS*** *p.Gly12Asp;* ***TP53*** *p.Ile251Phe;* ***ATM*** *p.Lys1744Asn*	
38.	***MET*** *p.Asp1028Tyr;* ***TP53*** *p.Glu271Lys*	***MET*** **and other genes (*N* = 2)**
39.	***MET*** *c.1200+1G>A;* ***MET*** *c.3082+1G>C;* ***TP53*** *p.Pro278Ser*	
40.	***PIK3CA*** *p.Glu545Lys;* ***PIK3CA*** *p.Glu726Lys;* ***FBXW7*** *p.Arg505Ser*	
41.	***PIK3CA*** *p.Glu545Lys****;*** ***VHL*** *p.Ile147Phe;* ***STK11*** *p.Pro281fs;* ***FLT3*** *p.Ala650fs;* ***TP53*** *p.Arg248Gln;* ***TP53*** *p.Trp146X*	***PIK3CA*** **and other genes (*N* = 2)**

Supplementary Table 4: Mutation frequency in all ethnicities by type of molecular testing

Mutations	All ethnicities All platforms (*N* = 515) *N* (%)	Next generation sequencing platforms				
		Ethnicity				
		Caucasian (*N* = 93) *N* (%)	Asian (*N* = 74) *N* (%)	South Asian (*N* = 8) *N* (%)	Black (*N* = 8) *N* (%)	Other (*N* = 6) *N* (%)
EGFR	269 (52.1)	25 (27)	29 (40)	3 (37.5)	2 (25)	2 (33.3)
ALK	39 (7.4)	5 (5.4)	1 (1.3)	–	–	–
KRAS	12 (2.3)	10 (10.8)	–	1 (12.5)	1 (12.5)	–
TP53	7 (1.4)	3 (3.2)	4 (5.3)	–	–	–
ERBB2	5 (1.0)	1 (1.1)	3 (4)	–	–	1 (16.7)
BRAF	2 (0.4)	1 (1.1)	1 (1.3)	–	–	–
PIK3CA	2 (0.4)	1 (1.1)	1 (1.3)	–	–	–
SMAD4	2 (0.4)	2 (2.2)	–	–	–	–
CTNNB1	1 (0.2)	1 (1.1)	–	–	–	–
AKT1	1 (0.2)	–	–	–	1 (12.5)	–
NRAS	1 (0.2)	1 (1.1)	–	–	–	
Multiple mutations	41 (7.9)	15 (16.1)	19 (25.3)	1 (12.5)	3 (37.5)	2 (33.3)
		EGFR and other (N = 8)<br>*KRAS and other (N = 2)*<br>*PIK3CA and other (N = 2)*<br>*ALK and other (N = 1)*<br>*BRAF and other (N = 1)*<br>*MET and other (N = 1)*	*EGFR and other (N = 14)*<br>*ALK (N = 2)*<br>*KRAS (N = 1)*<br>*ERBB2 (N = 1)*<br>*MET (N = 1)*	*EGFR (N = 1)*	*EGFR (N = 2)*<br>*ERBB2 (N = 1)*	*EGFR (N = 1)*<br>*KRAS (N = 1)*
None	133* (26.1)	27 (30.1)	15 (21.3)	3 (37.5)	1 (12.5)	1 (16.7)

*21 tumor samples of patients were tested only for *EGFR;* 68 tumor samples were tested only for *EGFR* and *ALK* (1 out of 68 was tested also for *ROS-1*); 43 patients' tumor samples were tested using multigene Next Generation Sequencing (NGS) assays: Sequenom MassARRAY (*N* = 22) and MiSeq Illumina (*N* = 21); 15 out of 43 available patients' tumor samples with no detected mutations when tested with NGS assays were tested for *ROS-1*.

Supplementary Table 5: Multivariable Cox proportional model for overall survival

Covariate	HR	95% CI	*p*-value	Overall *p*-value
**Patients with known mutations (*N* = 380)**				
Gender (M vs. F)	1.35	1–1.82	0.049	
Stage (II vs. I)	1.42	0.59–3.39	0.44	<0.0001
Stage (III vs. I)	2.70	1.42–5.15	0.0026	
Stage (IV vs. I)	5.06	2.89–8.85	<0.0001	
ECOG (1 vs. 0)	2.06	1.50–2.84	<0.0001	<0.0001
ECOG (2 vs. 0)	6.31	3.75–10.62	<0.0001	
ECOG (3/4 vs. 0)	16.99	8.06–35.81	<0.0001	
**Patients with and without mutation(s) (*N* = 380)**				
Gender (M vs. F)	1.33	0.99–1.79	0.063	
Stage (II vs. I)	1.19	0.49–2.85	0.7	<0.0001
Stage (III vs. I)	2.35	1.23–4.47	0.0094	
Stage (IV vs. I)	4.64	2.66–8.07	<0.0001	
ECOG (1 vs. 0)	2.14	1.55–2.94	<0.0001	<0.0001
ECOG (2 vs. 0)	5.81	3.44–9.80	<0.0001	
ECOG (3/4 vs. 0)	17.04	8.11–35.79	<0.0001	
Mutation (Y vs. N)	0.51	0.37–0.69	<0.0001	
**Patients with and without *EGFR* (*N* = 380)**				
Gender (M vs. F)	1.28	0.95–1.73	0.11	
Stage (II vs. I)	1.43	0.6–3.4	0.42	<0.0001
Stage (III vs. I)	2.74	1.45–5.16	0.0019	
Stage (IV vs. I)	5.11	2.96–8.85	<0.0001	
ECOG (1 vs. 0)	2.08	1.51–2.86	<0.0001	<0.0001
ECOG (2 vs. 0)	6.45	3.83–10.86	<0.0001	
ECOG (3/4 vs. 0)	17.00	8.16–35.40	<0.0001	
EGFR mutation (Y vs. N)	0.72	0.54–0.96	0.025	
**Patients with and without *ALK* (*N* = 380)**				
Gender (M vs. F)	1.47	1.09–2.00	0.012	
Stage (II vs. I)	1.13	0.47–2.73	0.79	<0.0001
Stage (III vs. I)	2.25	1.17–4.33	0.015	
Stage (IV vs. I)	4.58	2.61–8.03	<0.0001	
ECOG (1 vs. 0)	2.13	1.55–2.93	<0.0001	<0.0001
ECOG (2 vs. 0)	5.86	3.48–9.87	<0.0001	
ECOG (3/4 vs. 0)	14.75	6.97–31.22	<0.0001	
ALK mutation (Y vs. N)	0.31	0.15–0.64	0.0014	
**Patients with *EGFR* and *ALK* (*N* = 261)**				
Gender (M vs. F)	1.33	0.88–2.01	0.17	
Stage (II vs. I)	1.41	0.48–4.16	0.53	= 0.00013
Stage (III vs. I)	1.78	0.74–4.26	0.2	
Stage (IV vs. I)	4.11	1.91–8.86	0.00030	
ECOG (1 vs. 0)	1.92	1.28–2.89	0.0017	<0.0001
ECOG (2 vs. 0)	4.08	1.85–8.98	0.00048	
ECOG (3/4 vs. 0)	10.83	3.06–38.29	0.00022	
EGFR vs. ALK	2.69	1.28–5.64	0.0088	
